# Risk stratification for sudden cardiac death in nonischemic dilated cardiomyopathy: a moving target?

**DOI:** 10.3389/fcvm.2026.1766952

**Published:** 2026-05-20

**Authors:** Laura Bradel, Katherine Kovrizhkin, Harout Yacoub, Gioia Turitto

**Affiliations:** 1 Department of Medicine, NewYork-Presbyterian Brooklyn Methodist Hospital, Brooklyn, NY, United States; 2 Department of Medicine, Albert Einstein College of Medicine, New York, NY, United States; 3 Department of Medicine, Weill Cornell Medicine, New York, NY, United States

**Keywords:** cardiac MR (CMR), ICD (implantable cardioverter-defibrillator), non ischemic cardiomyopathy, SCD, ventricular arrhythmia

## Abstract

Risk stratification for sudden cardiac death (SCD) in non-ischemic dilated cardiomyopathy (NICM) and a left ventricular ejection fraction (LVEF) ≤35%. remains controversial. The value of a low LVEF alone is limited, as it cannot distinguish between arrhythmic and nonarrhythmic mortality. This led to further investigation into markers of electrical instability. Prior studies have evaluated noninvasive and invasive markers, including ambient arrhythmias, signal-averaged ECG, QT dispersion, T-wave alternans, heart rate variability, and programmed ventricular stimulation. None of these markers have consistently improved the predictive accuracy for SCD beyond LVEF. Cardiac magnetic resonance (CMR) imaging with late gadolinium enhancement (LGE) has emerged as a tool for substrate characterization, with myocardial fibrosis burden and LGE patterns associated with SCD, arrhythmic events, as well as appropriate therapies in patients with implantable cardioverter-defibrillators. Quantitative LGE thresholds and integrated CMR-based models may enhance SCD risk discrimination, although methodological heterogeneity, scanner-dependent quantification, and lack of randomized trials challenge the inclusion of CMR parameters in current guidelines. Emerging tiered approaches like the ReCONSIDER framework, combining noninvasive markers, CMR tissue characterization, and electrophysiologic testing to capture the heterogeneity of NICM, may also be of value. However, at the present time, no single imaging, clinical, or electrophysiologic marker has achieved guideline-level validation as an adjunct or a replacement for LVEF. Future studies may focus on standardizing CMR acquisition and quantification, prospective validation of multimodal risk models, and assessment of dynamic, serial biomarkers to establish a more accurate approach to SCD risk stratification in NICM.

## 1 Introduction

**Figure 1:**
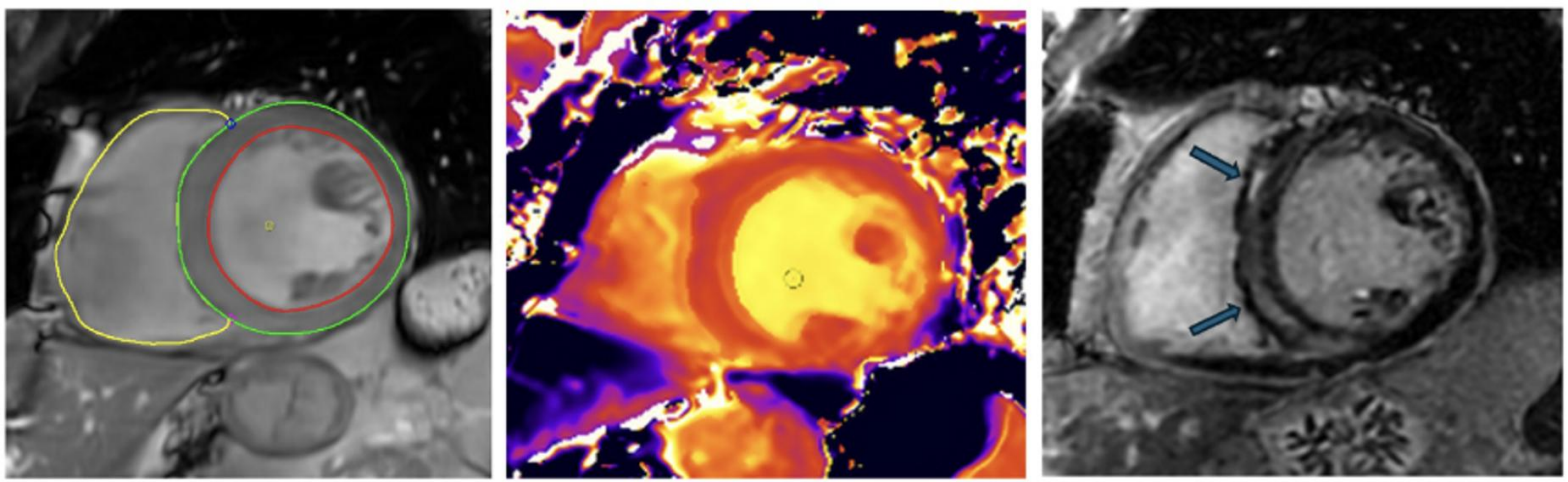
CMR images from a patient with dilated cardiomyopathy and non-sustained ventricular tachycardia. First image on the left: Cine short axis view. The short axis images are acquired form apex to base to quantify left ventricular/right ventricular volume and systolic function. T1 mapping images with septal increase in T1 values. LGE images showing mid septal scar. Scar burden is 4%.

Non-ischemic cardiomyopathies are myocardial disorders in which the heart muscle is structurally and functionally abnormal, in the absence of coronary artery disease, hypertension, valvular disease, or congenital heart disease (1). An optimal risk stratification for sudden cardiac death (SCD) in patients with non-ischemic dilated cardiomyopathy (NICM) and left ventricular ejection fraction (LVEF) ≤35% is still lacking (2) (Figure 1).

The main limitation of the current strategy is that a low LVEF may predict both sudden and non sudden cardiac death, thus decreasing its value to select candidates for implantation of a cardioverter-defibrillator (ICD) (2, 3). Multiple indices of electrical instability, such as spontaneous non-sustained ventricular tachycardia (NSVT) (4), the signal averaged ECG (5, 6), QT interval duration and dispersion (7), T-wave alternans (8, 9), heart rate variability (9, 10), inducible ventricular tachycardia/fibrillation (VT/VF) by programmed electrical stimulation (6, 9, 11) have been studied in combination with low LVEF. The ReCONSIDER study proposed a multifaceted strategy, combining noninvasive risk factors with programmed ventricular stimulation to identify high-risk NICM patients (12). However, none of the studied electrical markers significantly improved the predictive accuracy of low LVEF alone for SCD.

More recently, it was suggested that genetic factors may play a role in the pathogenesis and arrhythmic risk of NICM. Pathogenic variants are identified in approximately 20%–35% of patients with dilated cardiomyopathy (13). The 2023 European Society of Cardiology (ESC) guidelines incorporate genotype-specific risk factors into primary prevention ICD recommendations for patients with dilated cardiomyopathies, recognizing that patients with certain variants have a higher rate of major arrhythmic events than other causes of NICM regardless of LVEF (14).

Cardiac magnetic resonance (CMR) imaging with late gadolinium enhancement (LGE) has been evaluated as a predictor of arrhythmic events in recent literature. The presence of regional myocardial fibrosis and high scar burden was associated with cardiac mortality, SCD, and appropriate ICD therapy in patients with NICM (15–21). However, LGE guided ICD implantation has a limited role in the current guidelines due to the availability of the test, the heterogeneity in LGE acquisition and quantification, and the absence of randomized trials testing LGE-guided ICD implantation.

The current literature shows the limitations of LVEF based selection of ICD placement and the need for a more integrated and substrate-based informed approach**.** The lack of a validated or widely accepted framework for SCD risk stratification in patients with NICM emphasizes the need for continued prospective studies.

## 2 Traditional left ventricular ejection fraction strategy

Current guidelines recommend ICD implantation for primary prevention of SCD in patients with NICM and LVEF of ≤35% and New York Heart Association (NYHA) class II–III symptoms despite adherence to guideline-directed therapy (22, 23). The DANISH trial did not demonstrate a statistically significant reduction in all-cause mortality in the overall cohort, despite a significant reduction in sudden cardiac death (HR 0.50) and evidence of age-dependent benefit, with greater survival advantage observed in younger patients (24, 25). On one hand, findings from recent registry-based observational studies (26, 27) and meta-analyses that included the results from DANISH trial (28, 29) supported current guideline recommendations, whereas newer observational cohort studies and *post hoc* analyses (30–32) supported the conclusions of the DANISH investigators. These findings have driven further research for a more accurate risk stratification beyond LVEF (Table 1).

**Table 1:** Current algorithms for risk stratification for sudden cardiac death in patients with non ischemic dilated cardiomyopathy.

Strategy	Key Concept	Main Components	Key Findings	Pitfalls/Shortcomings
Left Ventricular Ejection Fraction Strategy	LVEF-based ICD implantation for primary prevention in NICM, LVEF ≤35% with NYHA II–III on medical therapy.	Resting LVEF by echo or CMR and NYHA functional class.	Forms the basis of guideline ICD indications. However, in the DANISH trial ICD implantation in NICM did not reduce all-cause mortality	Poor specificity for arrhythmic events. Many patients with low LVEF never receive appropriate ICD therapy. Competing HF and non-cardiac deaths reduce ICD benefit. LVEF reflects heart failure more than SCD.
Clinical Risk Factors and Invasive Electrophysiology Testing Strategy	Multiple step risk assessment combining non-invasive clinical risk markers with programmed ventricular stimulation to identify patients with inducible VT/VF.	Non-invasive risk factors, history of unexplained syncope, LVEDD >60 mm, presence of LGE, >30 PVCs/hour, NSVT, late potentials, prolonged QTc, T-wave alternans, impaired heart rate variability, followed by PVS using up to 3 extrastimuli from 2 RV sites ± *β*-agonist.	ReCONSIDER applies this two-step algorithm to NICM patients with LVEF ≤35% and 35–50%**,** classifying them into six groups according to LVEF, non-invasive risk factors, and PVS inducibility. ICDs are given to guideline-eligible EF ≤35% patients and to EF 35–50% patients with positive PVS. The design aims to more precisely select truly high-risk patients for ICD therapy.	Evidence is currently design level only without current outcome data.
Echocardiographic and Strain-Based Markers Strategy	Implementation of strain as a parameter for myocardial deformation and function for detection of myocardial dysfunction associated with VA and SCD in NICM.	Global Longitudinal Strain (GLS) via speckle-tracking, ventricular mechanical dispersion.	Impaired GLS and mean longitudinal strain have been found to be independent predictors of cardiac death, heart transplantation, and aborted SCD. Strain is found to be superior in risk prediction compared to NYHA, EF, and LGE.	Lack of standardized strain thresholds limits generalizability.
CMR LGE Qualitative Presence and Pattern Strategy	Characterize the presence, location and pattern of myocardial fibrosis via LGE to detect arrythmia generating foci in NICM.	Qualitative assessment of LGE presence, pattern, and distribution	LGE is a sensitive predictor of future events in patients with NICM and reduced LVEF who have ICD for primary prevention. Presence of LGE in NICM is an independent predictor for VA, SCD, HF hospitalization, and mortality. Patients with LGE-negative NICM have significantly lower arrhythmia risk. Heterogeneous LGE patterns are associated with higher VA burden. Septal midwall LGE is associated with increased risk for SCD and appropriate ICD therapy. Basal LGE was a stronger predictor for monomorphic VT in NICM patients with ICD. However, other studies show that LGE was associated with arrythmia risk regardless of pattern.	Lack of standardized cutoffs. Variance in methodology for defining scar presence. Contradictory findings concerning role of extent, localization, and pattern.
Quantitative LGE and Integrated CMR-Based Risk Model Strategy	Multimodal, CMR-based risk models integrating quantitative measures of myocardial fibrosis and CMR parameters.	Quantitative LGE burden (% of LV mass), scar mass and border-zone mass, scar heterogeneity, extracellular volume (ECV) and native T1 mapping for diffuse fibrosis, LV geometry and remodeling markers (LVEDVi, LV sphericity), CMR-derived strain	LGE extent is associated with ventricular arrythmias, SCD, HF hospitalization, transplant and mortality. In patients with NICM and primary prevention ICDs, significant LGE burden (LGE ≥2%) is predictive of appropriate device therapies but not all-cause mortality/LVAD/transplant.	LGE quantification requires specific software and expertise. There exists variance in cut off values for “high risk” LGE burden.
Stand Alone T1 Mapping Strategy	Native T1 mapping; post-contrast T1 mapping; extracellular volume (ECV) quantification; pixel-wise quantitative myocardial tissue characterization	T1 and ECV mapping detect diffuse interstitial fibrosis, providing quantitative parametric assessment of myocardial microstructure.	Detects diffuse interstitial fibrosis not visible on LGE imaging. Native T1 and ECV values correlate strongly with histologic content, allowing earlier detection of myocardial disease activity. Elevated native T1 and increased ECV predict heart failure and prognosis independent of LGE	Technique is limited by field-strength dependence**,** sequence variability, vendor differences, heart-rate sensitivity, and need for hematocrit calibration, all of which complicate inter-center comparability and standardization of prognostic cutoffs. Reference ranges differ by scanner and institution, and prognostic thresholds in NICM have not been validated in prospective trials.

LVEDD, LV end-diastolic diameter; PVC, premature ventricular complexes; PVS, programmed ventricular stimulation.

## 3 Clinical risk factors and invasive electrophysiology testing strategy

Multiple markers for electrical instability have been evaluated, but none have complemented LVEF in the current guidelines (22, 23). The ReCONSIDER study is an example of a new approach. It was a prospective multicenter observational trial in NICM that proposed a two-step algorithm by screening with multiple non-invasive risk factors, followed by programmed ventricular stimulation to assess inducibility of ventricular tachycardia/fibrillation (VT/VF). This combined risk stratification formed six subgroups with ICD allocation based on the results. The rationale was to address the heterogeneity of dilated cardiomyopathy and the inadequacy of LVEF alone, by combining substrate assessment with noninvasive arrhythmic markers and the demonstration of inducible ventricular arrythmias (12). The trial remains ongoing, and only the study rationale and methodological framework have been published. Clinical outcomes assessing the impact of CMR-guided risk stratification on sudden cardiac death or ICD utilization in NICM have not yet been reported (12).

## 4 Echocardiographic and strain based markers strategy

Global longitudinal strain, as assessed by echocardiogram or CMR, has been associated with cardiac mortality and major arrhythmic events (33–37). A major advantage of echocardiography, when compared to CMR, is its cost-effectiveness and availability. Limitations of this assessment include the fact that different software and techniques elicit different absolute values and cut-offs. Furthermore, the specificity of these indices for SCD is still unproven. Strain-based risk markers have been evaluated in non-ischemic heart failure populations with LVEF ≤35% using SCD associated endpoints. In a cohort of 401 non-ischemic heart failure patients with LVEF <35%, inferior wall longitudinal strain below the median was independently associated with a composite outcome including SCD, sustained ventricular arrhythmia, resuscitated cardiac arrest, and appropriate primary-prevention ICD therapy during a median follow up of 4 years, whereas global longitudinal strain and LVEF were not independently associated with outcome (36). On the other hand, a prospective study that followed 94 patients with NICM for 22 months showed that global longitudinal strain had a greater area under the curve than LVEF to identify arrhythmic events in receiver operating characteristic curve (37).

## 5 CMR LGE qualitative presence and pattern strategy

A range of CMR parameters has been associated with an increased risk of SCD. Applicable examples include LGE, T1 relaxation times, and myocardial strain (38). A large, retrospective cohort study included 1,165 patients with NICM who underwent CMR with LGE at 2 tertiary referral centers (39). The combined arrhythmic endpoint included appropriate ICD therapies, sustained VT, resuscitated cardiac arrest, and SCD. Mean follow up was 36 months. LGE was an independent and strong predictor of the arrhythmic endpoint (*p* < 0.001). This association was consistent across all strata of LVEF. Epicardial LGE, transmural LGE, and combined septal and free-wall LGE were all associated with heightened risk. A simple algorithm combining LGE and 3 LVEF strata (i.e., ≤20%, 21% to 35%, >35%) was significantly superior to LVEF with the 35% cutoff (*p* < 0.001) and reclassified the arrhythmic risk of 34% of patients with NICM. LGE-negative patients with LVEF 21% to 35% had low risk (annual event rate 0.7%), whereas those with high-risk LGE distributions and LVEF >35% had significantly higher risk (annual event rate 3%; *p* = 0.007) (39). A meta-analysis of left ventricular mid wall LGE in NICM (7 studies, 1,827 patients) showed that mid wall LGE predicts all-cause mortality and cardiovascular death and combined SCD/aborted SCD events independent of LVEF above or below 35%. The high negative predictive value for SCD suggests that absence of mid wall LGE identifies a low-risk subgroup (16). Another meta-analysis of primary-prevention ICD in NICM patients (11 studies, 1,652 patients) found that LGE presence was associated with appropriate device therapy and cardiac death, with high sensitivity and only moderate specificity. LGE was a sensitive marker for future appropriate ICD therapies, but many LGE-positive patients would never receive therapy. LGE absence was associated with low event rates, but it moderate specificity would be insufficient to deny an ICD (40). A broader CMR review showed that LGE was an independent predictor of ventricular arrhythmias and SCD, but there were contradictory findings about the prognostic role of LGE extent, location, and pattern, due to technical heterogeneity in how LGE was quantified. Variation in LGE acquisition, thresholding, and post-processing limits generalizability and makes universal cut-offs hard to define (38). Further study are need to definitely prove that an LGE algorithm incorporating both LGE presence/pattern as well as LVEF may provide a more nuanced assessment of arrhythmic risk in patients with DCM (41). In the DERIVATE registry, 1,384 patients with NICM, chronic systolic heart failure, and LVEF <50% undergoing evaluation for primary SCD prevention were followed for a median of 959 days, during which major arrhythmic adverse events occurred in 9.2% of cases. In the multivariate analysis, LVEF, male gender, and presence and location of midwall LGE were independent predictors of major arrhythmic events. The LGE term modeled as a weighted measure reflecting scar distribution. The CMR-based DERIVATE Risk Score 2.0 provided incremental prognostic value and improved reclassification compared with the traditional LVEF <35% threshold (net reclassification improvement 55%, *P* < 0.001). This finding describes the importance of incorporating LGE pattern distribution into contemporary NICM arrhythmic risk assessment (42).

## 6 Quantitative LGE and integrated CMR based risk model strategy

Recent studies have moved toward quantitative thresholds and integrated models for LGE. Zhou et al. studied 1,272 NICM patients with LVEF ≤35% and developed a risk model incorporating age, family history of SCD, NT-proBNP, and LVEF (17). In his risk model, LGE ≥7.5% of LV mass was an independent predictor of SCD/aborted SCD over a mean follow up of 86 months. Age, family history of SCD, NT-proBNP, LVEF and LGE ≥7.5% were associated with SCD/aborted SCD. Compared with LGE < 7.5%, patients with LGE≥7.5% and LVEF≤20% had a 7.12-fold higher risk of experiencing SCD in competing Cox analysis (annual event rate, 4.8%). Left atrial volume index (LAVi ≥68.3 mL/m^2^) was a specific predictor of heart failure death/transplant to help separate arrhythmic from heart failure risk (17). Kiang et al. studied 344 DICM patients with ICDs placed for primary prevention and pre-implant CMR by dividing LGE burden at 2% of LV mass (15). LGE ≥2% independently predicted appropriate ICD therapies but did not predict all-cause mortality. This suggested that LGE may be more arrhythmia specific than LVEF (15). A 2025 meta-analysis of LGE and left ventricular reverse remodeling in NICM (13 studies, 1,092 patients) found that absence of LGE led to reverse remodeling with medical therapy regardless of baseline LVEF (43). This may suggest patients with negative LGE may benefit more from medical therapy and less from primary-prevention ICDs, but this remains unknown as SCD endpoints were not studied. There are pitfalls to the CMR model. Quantitative LGE thresholds (≥2%, ≥7.5%) are scanner, sequence, and processing dependent and may not transfer across platforms. Risk models do not provide a rule for not placing an ICD as SCD events in low-risk patients may be low but are still present (17). No single CMR parameter currently discriminates SCD from non-SCD. CMR risk predictors have not yet been used in trials on which current ICD guidelines are based. An analysis from Hammersley et al. suggests that myocardial scar heterogeneity may provide prognostic information in patients with NICM. The concept of fibrosis entropy is a quantitative CMR-derived measure of scar heterogeneity. This was evaluated and demonstrated that higher fibrosis entropy was associated with an increased risk of life-threatening ventricular arrhythmias particularly in patients with NICM. In multivariable models, fibrosis entropy remained independently associated with arrhythmic events after adjustment for conventional risk markers, supporting the hypothesis that scar complexity, and spatial heterogeneity may represent a substrate for ventricular arrhythmias that can lead to sudden cardiac death than scar burden alone (44).

## 7 Standalone T1-mapping strategy

The T1 mapping CMR technique is used for comprehensive myocardial tissue characterization, which can offer sensitivity to diffuse interstitial fibrosis that would normally be missed by LGE (Figure 1). T1 mapping provides a quantitative, pixel-wise assessment of myocardial relaxation properties that reflect changes in the extracellular matrix, edema, and inflammation which is unlike LGE as it identifies focal replacement fibrosis (45). Extracellular volume (ECV) quantification derived from pre- and post-contrast T1 measurements further improves the detection of diffuse matrix expansion and corresponds with histologic fibrosis burden (46, 47). Native T1 and ECV mapping can improve diagnostic accuracy in NICM, identify early disease before overt remodeling occurs, and offer prognostic value more so than LGE (45). These findings support incorporation of T1 mapping into CMR strategies and its potential to surpass key limitations of LGE-only assessment (46). T1 mapping and ECV have demonstrated prognostic value in NICM/DCM, although the strongest evidence relates to heart failure progression and mortality rather than SCD.

## 8 Genotype based risk stratification

Genotype-specific risk is explicitly incorporated into contemporary guideline-based risk stratification. The 2023 ESC Cardiomyopathy Guidelines emphasize that the patient's genotype should be considered in the estimation of SCD risk. ICD implantation should be considered in patients with DCM with a genotype associated with high SCD risk and LVEF >35% in the presence of additional risk factors such as NSVT, increased ventricular ectopic beats, male sex, significant LGE, specific gene variant, both of which are a Class IIa recommendation. The ESC guidelines recognize specific variants including Lamin A/C, Filamin, and Desmoplakin as high risk arrhythmic genotypes, for which exceptions to conventional LVEF cut offs may be appropriate in clinical decision-making (14).

## 9 Discussion

Attempts at SCD risk stratification in NICM have progressively shifted from LVEF-based selection to multifactorial approaches integrating imaging, clinical markers, and electrophysiologic data. The ReCONSIDER study added an important framework creating a tiered, non-invasive risk factor to invasive electrophysiology approach that addresses major limitations of prior methods and incorporates LGE as a main substrate marker for SCD. No current method has achieved guideline level acceptance as a replacement for LVEF in determining ICD candidacy. While markers such as genotype and CMR with LGE are incorporated into current guidelines as adjuncts to refine risk stratification, they have not replaced LVEF as the primary determination of eligibility of an ICD. CMR, with LGE, may substantially improve our ability to risk stratify patients with NICM, but because of heterogeneity in methods, competing heart failure mortality, and lack of prospective ICD trials, it has not yet fully solved the problem of which patients should receive an ICD. Further standardization of CMR acquisition and LGE quantification is essential, as methodological heterogeneity hampers reproducibility across hospital centers. Novel quantitative CMR techniques such as native T1 and ECV mapping may further enhance substrate characterization by detecting diffuse fibrosis not captured by LGE alone, offering an important future direction for refining SCD risk stratification in NICM. The performance of strain and other echo-based metrics depends on unity across imaging platforms as well. Future work should focus on validating multiparametric prediction models**,** such as those incorporating both quantitative LGE and clinical variables, and evaluating whether serial assessment of fibrosis, strain, or arrhythmia burden can support dynamic risk stratification rather than reliance on static, baseline metrics such as LVEF. Several randomized controlled trials are currently recruiting to bridge this gap in evidence. The results of these trials may prove crucial in pivoting away from LVEF based risk stratification when it comes to offering ICD therapy in NICM (47–49).
