# Supplementary figures and images for: Risk stratification for sudden cardiac death in nonischemic dilated cardiomyopathy: a moving target?
Source: Front Cardiovasc Med. 2026 May 20;13:1766952. doi: 10.3389/fcvm.2026.1766952 (PMC13229721; doi:10.3389/fcvm.2026.1766952)

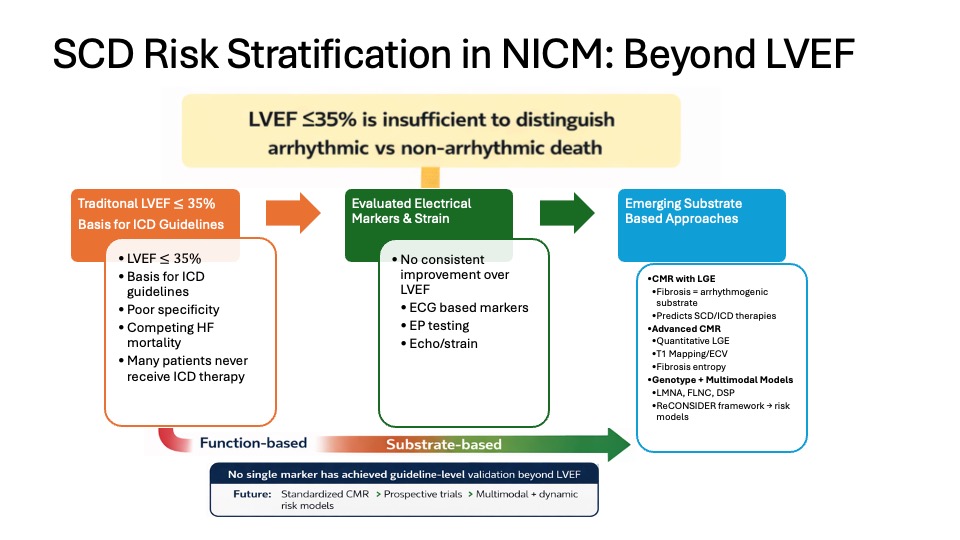

Supplement: Supplementary file 1 [file Image1.jpeg]
